# Novel Nomogram for Preoperative Prediction of Early Recurrence in Intrahepatic Cholangiocarcinoma

**DOI:** 10.3389/fonc.2018.00360

**Published:** 2018-09-04

**Authors:** Wenjie Liang, Lei Xu, Pengfei Yang, Lele Zhang, Dalong Wan, Qiang Huang, Tianye Niu, Feng Chen

**Affiliations:** ^1^Department of Radiology, The First Affiliated Hospital, College of Medicine, Zhejiang University, Hangzhou, China; ^2^Department of Hepatobiliary and Pancreatic Surgery, The First Affiliated Hospital, College of Medicine, Zhejiang University, Hangzhou, China; ^3^Institute of Translational Medicine, College of Medicine, Zhejiang University, Hangzhou, China; ^4^Department of Radiation Oncology, Sir Run Run Shaw Hospital, College of Medicine, Zhejiang University, Hangzhou, China; ^5^Collaborative Innovation Center for Diagnosis and Treatment of Infectious Diseases, The First Affiliated Hospital, College of Medicine, Zhejiang University, Hangzhou, China; ^6^Key Lab of Combined Multi-Organ Transplantation, Ministry of Public Health, The First Affiliated Hospital, College of Medicine, Zhejiang University, Hangzhou, China

**Keywords:** intrahepatic cholangiocarcinoma, recurrence, MRI, radiomics, machine learning

## Abstract

**Introduction:** The emerging field of “radiomics” has considerable potential in disease diagnosis, pathologic grading, prognosis evaluation, and prediction of treatment response. We aimed to develop a novel radiomics nomogram based on radiomics features and clinical characteristics that could preoperatively predict early recurrence (ER) of intrahepatic cholangiocarcinoma (ICC) after partial hepatectomy.

**Methods:** A predictive model was developed from a training cohort comprising 139 ICC patients diagnosed between January 2010 and June 2014. Radiomics features were extracted from arterial-phase image of contrast-enhanced magnetic resonance imaging. Feature selection and construction of a “radiomics signature” were through Spearman's rank correlation and least absolute shrinkage and selection operator (LASSO) logistic regression. Combined with clinical characteristics, a radiomics nomogram was developed with multivariable logistic regression. Performance of the nomogram was evaluated with regard to discrimination, calibration, and clinical utility. An independent validation cohort involving 70 patients recruited from July 2014 to March 2016 was used to evaluate the utility of the nomogram developed.

**Results:** The radiomics signature, consisting of nine features, differed significantly between ER patients and non-ER patients in training and validation cohorts. The area under the curve (AUC) of the radiomics signature in training and validation cohorts was 0.82 (confidence interval [CI], 0.74–0.88) and 0.77 (95% CI, 0.65–0.86), respectively. The AUC of the radiomics nomogram combining the radiomics signature and clinical stage in the two cohorts was 0.90 (95%CI, 0.83–0.94) and 0.86 (95% CI, 0.76–0.93), respectively. Decision curve analysis confirmed the clinical usefulness of the radiomics nomogram.

**Conclusion:** The non-invasive radiomics nomogram developed using the radiomics signature and clinical stage could be used to predict ER of ICC after partial hepatectomy.

## Introduction

Cholangiocarcinoma is the second most aggressive primary tumor of the liver after hepatocellular carcinoma (HCC) (1). These tumors can be classified into “intrahepatic cholangiocarcinoma” (ICC) and “extrahepatic cholangiocarcinoma” (ECC) according to the pathogenic sites involved.

As a lethal primary cancer arising from malignant transformation of the bile-duct epithelium and hepatocyte transdifferentiation, ICC accounts for 10–25% of all primary liver cancers worldwide (1). Resection is the principal treatment for ICC. Preoperative evaluation of ICC is commonly based on the 7th edition of Tumor, Node, and Metastasis staging of the American Joint Committee on Cancer (AJCC)/International Union Against Cancer system (2).

Tumor stage is predictive of the risk of tumor relapse, but postoperative early recurrence (ER) for individual patients with ICC varies distinctly even within patients of identical stage, often causing treatment failure and death from ICC (3). In fact, even though the 5-year survival of ICC after surgery is 39.5%, the median disease-free survival is only 12 months (4). The morbidity and mortality of ICC have increased annually over the past 30 years, especially in Eastern Asia (3). It remains a challenge for clinicians to identify reliably those patients at high risk for ER and hinders the decision-making process of individualized treatment for patients with ICC.

Recent studies have shown that clinical factors can play important parts in determining appropriate treatment and associated outcomes. A nomogram model focused on these factors has been established for risk stratification and prediction of clinical behavior (5). Combining clinical factors into a statistically predictive model can improve personalized prognosis upon AJCC-based tumor staging. However, the information related to the spatial and temporal intra-tumor heterogeneities within ICC can influence the effectiveness of stratifying ER risk substantially. Subsequent “tailoring” of treatments remains limited. This has led clinicians to seek safe, efficacious, and novel methods to identify additional characteristics of ICCs that can improve the prediction of clinical behavior.

The emerging discipline of “radiomics” has helped generate high-dimensional, quantitatively mineable signatures extracted from medical imaging to evaluate (non-invasively) tumor phenotypes (6–8), tumor-cell proliferation, liver function, and patient prognosis (9). Radiomics carries the potential to enhance the precision of decision-making during the diagnosis, treatment, therapeutic evaluation, and prognostic evaluation of cancers (10–14). Furthermore, by analyzing multiple image features, these extracted radiomics signatures could be used to characterize intra-tumor heterogeneity, which may improve the predictive accuracy of the prognosis of cancer treatment. A widely recognized, accurate preoperative model predicting ER of ICC to guide individualized treatment recommendation is lacking.

We developed and validated a “radiomics signature” that could stratify ICC patients undergoing resection according to ER risk. The image features and radiomics signature were derived from contrast-enhanced magnetic resonance imaging (MRI) arterial-phase images of patients with ICC. We also investigated the gain in accuracy of the radiomics nomogram model by incorporating the radiomics signature and clinical risk factors for preoperative prediction of ER of ICC.

## Materials and methods

### Patients and MRI acquisition

This retrospective study was approved by the Institutional Review Board (IRB) of the First Affiliated Hospital, College of Medicine, Zhejiang University (Zhejiang, China). A waiver of written informed consent was obtained from the IRB.

The inclusion criteria for patients in this study were: (i) an ICC that was resected with a confirmed pathologic diagnosis; (ii) a contrast-enhanced MRI was carried out ≤ 4 weeks before resection; (iii) clinical data and follow-up data were complete. The exclusion criteria were: (i) ICC was confirmed by biopsy; (ii) the disease was confirmed to be combined HCC plus cholangiocarcinoma; (iii) the patient was treated before contrast-enhanced MRI. The patient-recruitment process is shown in Supplementary Data I.

Two independent datasets were used in this study. The training cohort used to construct the predictive model involved 139 ICC patients diagnosed between January 2010 and June 2014. This cohort consisted of 85 males and 54 females (range, 44–86 years; mean, 59.54 ± 9.75 years). The independent validation cohort, which was used to test the predictive model, comprised 70 patients (46 males and 24 females; range, 40–80 years; mean, 59.70 ± 9.02 years) diagnosed between July 2014 and March 2016. Imaging data were archived within the Picture Archiving and Communication System at the First Affiliated Hospital. The reliability of this study was evaluated by calculating a power of the test based on sample sizes and ER in the two cohorts (15).

Two surgeons assessed the clinical characteristics of the included patients independently. The clinical characteristics (sex, age, cholelithiasis (presence or absence), hepatitis (presence or absence), liver cirrhosis (presence or absence), affected site (left lobe or right lobe), maximum diameter of tumor, tumor number, clinical stage (I/II or III/IV), surgical margin (positive or negative), degree of tumor differentiation) were gathered and collated from the electronic medical-record system. The clinical stage was determined based on the 7th edition of the AJCC staging system (3). The patients underwent serial imaging approximately every 3 months after surgery to detect ER. The condition and time of recurrence were recorded during clinical and telephone follow-up. These were confirmed by definite pathology or confident imaging diagnosis according to a study on HCC recurrence (16). Patients who suffered recurrence within 1 year of their partial hepatectomy were classified as the ER group, whereas those without recurrence or suffering recurrence after 1 year of surgery were defined as the non-ER group.

Laboratory examination results (serum levels of alanine transaminase (ALT), aspartate transaminase (AST), carbohydrate antigen 19-9 (CA19-9), carcinoembryonic antigen (CEA) were acquired via routine blood tests < 2 weeks before surgery. The threshold values for ALT, AST, CA19-9, and CEA used here were 35, 50, 37 μg/mL, and 5 ng/mL, respectively.

Patients underwent imaging on a 3.0-T MRI scanner (GE Medical Systems, Milwaukee, WI, USA) using a breath-hold, fat-suppressed three-dimensional fast-spoiled gradient-recalled echo sequence (liver acceleration volume acquisition) (17). The acquisition parameters for MRI were: repetition time (TR) of 2.8 ms; echo time (TE) of 1.3 ms; reverse time of 5 ms; flip angle of 10°; field of view of 380 × 304 mm; bandwidth of 390.6 kHz; image resolution of 0.78 × 0.78 × 5 mm. Each patient was injected with a dose of 0.1 mmol/kg of gadopentetate dimeglumine through the median cubital vein via a high-pressure injector (2.5 mL/s). The arterial phase, portal-vein phase, and delayed phase were imaged at 14, 55, and 120 s after injection, respectively.

### Region of interest (ROI) segmentation, feature extraction, and building of a radiomics signature

**Figure 1:**
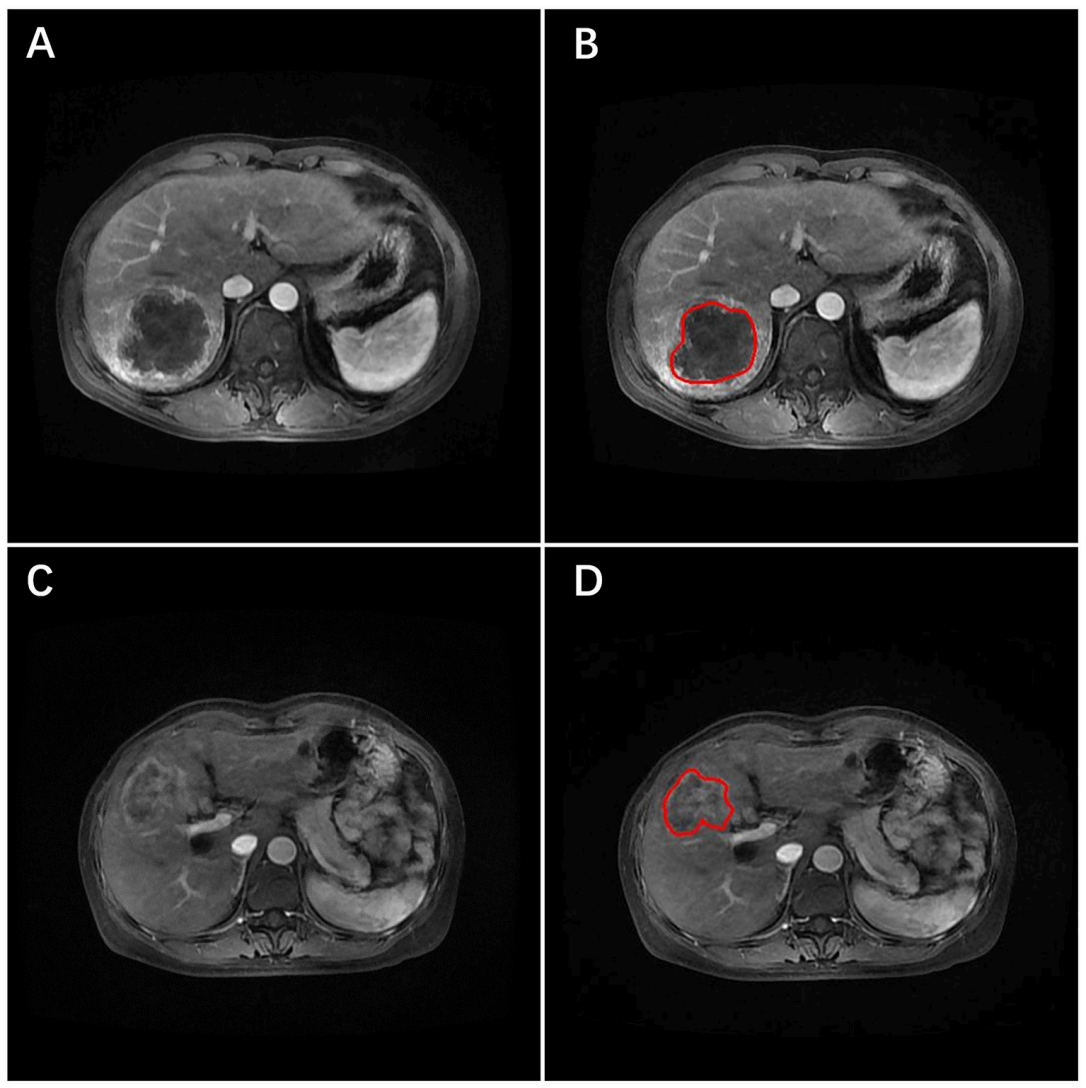
Arterial-phase contrast-enhanced MRI images of two patients with ICC. The tumor was identified, and the region of interest (ROI) placed (red line) on the images. **(A,B)** The primary image and the ROI (red line) marked image for one patient developed early recurrence. **(C,D)** The primary image and the ROI marked (red line) image for the other patient did not develop early recurrence.

The ROIs of tumors were segmented manually on ITK-SNAP v3.6.0 (www.itksnap.org) (18) by two radiologists with extensive clinical experience in making imaging diagnoses of the abdomen. An example of definition of ICC volume of two patients is given in Figure 1. The predictive model was constructed based on the radiomics features extracted from the contours identified by the first radiologist. As an internal validation, the reproducibility of these contours was assessed using the radiomics features extracted from the contours identified by the second radiologist.

Radiomics features can characterize the heterogeneity and complexity within tumors using a large set of quantitative features. The pre-processing procedure (i.e., image resampling and gray-level quantization) was undertaken before feature extraction. Radiomics features were extracted from ROIs contoured by the two radiologists (11). Based on the preoperative arterial-phase images on MRI, three groups of image features were extracted: (i) 6 histogram statistical features; (ii) 53 texture features; (iii) 408 wavelet features. A detailed description of these features is provided in Supplementary Data II.

Due to the large number of radiomics features and relatively small size of the patient dataset, feature selection was essential to deliver the optimal predictive features and to avoid over-fitting. Feature selection was carried out in two steps based on the training cohort. First, Spearman's rank correlation coefficients were calculated to examine the internal correlation between individual features. Redundant features with linear correlation coefficients >0.95 were removed (19). Second, a least absolute shrinkage and selection operator (LASSO) logistic regression analysis was used to identify the most ER-related features (19, 20). Thus, the radiomics signature was established by linear combination of the selected features weighted by the corresponding LASSO coefficients. A radiomics score was obtained for each patient using the radiomics signature.

The performance of the radiomics signature was reported using the receiver operating characteristic curve (ROC) and area under the ROC curve (AUC) in the training cohort and independent validation cohort. These values ranged from 0.50 (prediction accuracy was the same as a random guess) to 1.00 (prediction result was 100% correct). Details of the LASSO algorithm can be found in Supplementary Data III. The formula for the radiomics signature is presented in Supplementary Data IV.

### Development and validation of a radiomics nomogram model

Clinical characteristics (sex, age, cholelithiasis, hepatitis, liver cirrhosis, tumor diameter, tumor number, clinical stage, blood tests) were analyzed with a Mann-Whitney *U*-test to examine the statistical difference between the ER and non-ER groups (13). The combination of the developed radiomics signature with different clinical characteristics was tested using multivariable logistic regression. The backward search method using the Akaike information criterion (AIC) score was employed to select the optimal combination. This strategy assessed the quality of the model developed with comprehensive consideration of the influences of the binomial deviance and the number of variables in the selection process (13). The model with the lowest AIC score was selected as the optimal model. The ROCs and AUCs among different combinatorial models were compared through a Delong test (21), with a significance level set at 0.05. Finally, a radiomics nomogram model was developed based on multivariate logistic regression.

The calibration and discrimination performances of the radiomics nomogram model were tested in the training and validation cohorts. Calibration performance was assessed by the calibration curve, which described the agreement between the predicted and observed risks of ER. A Hosmer–Lemeshow (H–L) test (22) was used to evaluate the goodness-of-fit of the nomogram model. The discrimination performance was also measured with an AUC. Internal validation was completed using the cohort of 209 patients segmented by the second radiologist. Independent validation was carried out using the independent validation cohort of 70 patients segmented by the first radiologist.

The clinical utility of the radiomics nomogram model was conducted with a decision curve analysis (DCA) in the internal and independent validation cohorts. The net benefit was determined by calculating the difference between the “true” positive rate and weighted false-positive rate across different threshold probabilities in the validation set (23, 24). Specifically, the weighing factor was the specific value of the threshold probability divided by 1 minus the threshold probability. A high net benefit suggested a higher true positive rate and relatively low false-positive rate. The decision curve was generated by plotting the net benefit against the threshold probability across the range of 0 to 1. DCA for the radiomics signature was also plotted.

### Statistical analysis

Statistical analyses were carried out on R v3.4.1 (www.Rproject.org) and MedCalc v15.2.2 (www.medcalc.org). LASSO logistic regression analyses, plotting of nomograms and calibration curves, H–L test, ROC and AUC, and DCA were performed on the packages “glmnet,” “rms,” “generalhoslem,” “pROC,” and “dca.R,” respectively. The reported significance levels are two-sided and set at 0.05.

## Results

### Basic features

**Figure 2:**
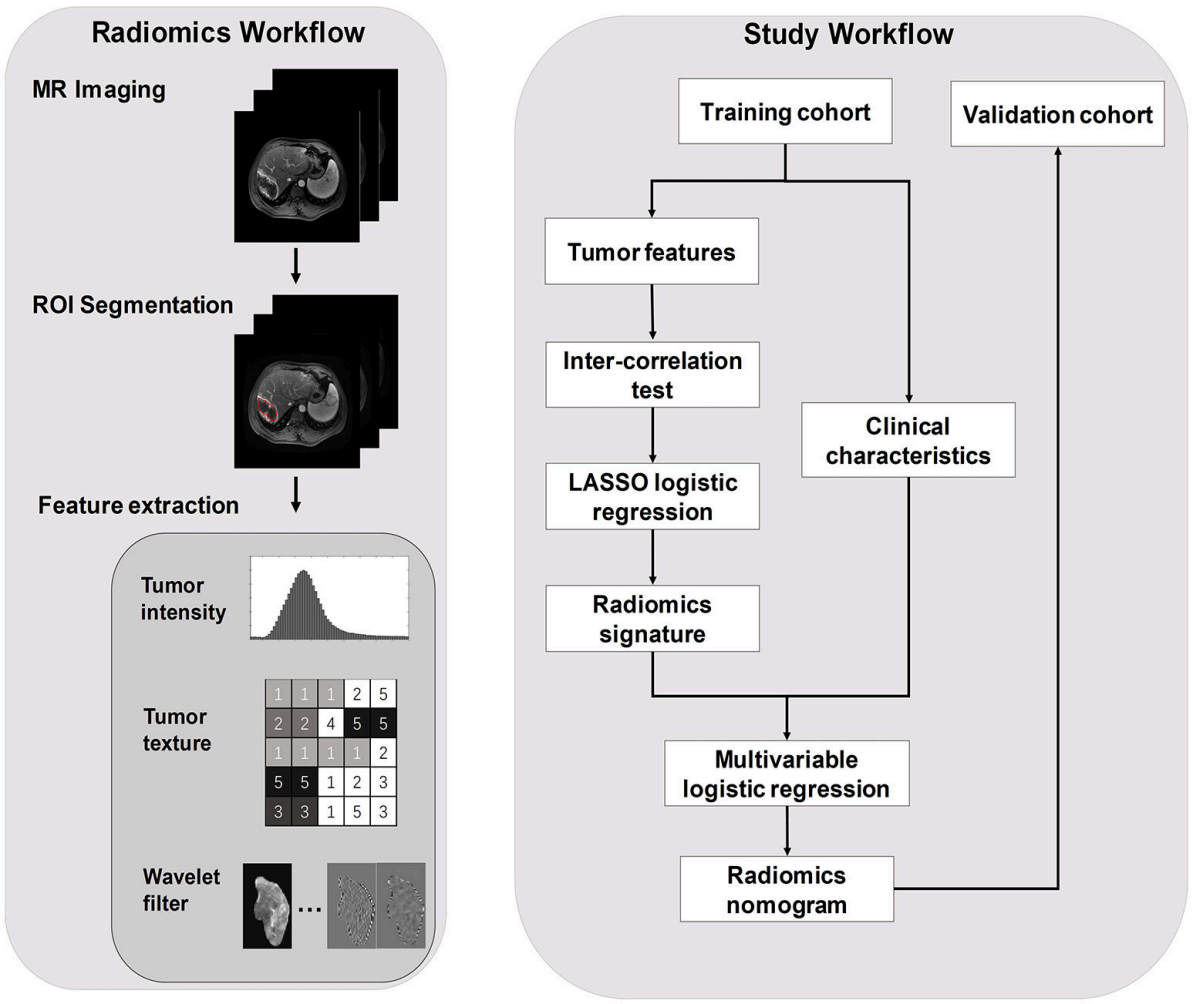
The radiomics workflow and study workflow.

The workflow of the radiomics nomogram model and flowchart of our study are presented in Figure 2.

### Clinical characteristics

Clinical characteristics in the training and validation cohorts are summarized in Table 1. There was no significant difference in the ER rate between training and validation cohorts (85/139 vs. 48/70, *p* = 0.29). The ER cases were intrahepatic alone (109, 81.96%), extrahepatic alone (17, 12.78%), or both (7, 5.26%). The position of extrahepatic recurrence was lymph node (13 cases), peritoneal and omental (4 cases), lung (3 cases), or other (4 cases).

**Table 1:** Clinical factors and radiomics score of the ER and Non-ER groups in two cohort.

	**Training cohort**			**Independent validation cohort**		
**Characteristic**	**ER (*n* = 85)**	**Non-ER (*n* = 54)**	***P***	**ER (*n* = 48)**	**Non-ER (*n* = 22)**	***P***
Age (years)	58.88 ± 9.14	60.57 ± 9.85	0.3042	59.69 ± 11.24	59.73 ± 8.32	0.6082
Gender (male: female)	51:34	34:20	0.7286	32:16	14:8	0.8076
Location (left: right)	49:36	29:25	0.6518	29:19	8:14	0.6512
Multiple (no: yes)	67:18	49:5	0.0682	34:14	19:3	0.1265
Maximum diameter (cm)	6.23 ± 2.63	4.54 ± 2.10	0.0001^*^	6.22 ± 2.23	4.09 ± 2.00	0.0003^*^
Hepatitis (no: yes)	64:21	34:20	0.1325	33:15	12:10	0.2740
Cirrhosis (no: yes)	76:9	50:4	0.5336	44:4	20:2	0.9196
Cholelithiasis (no: yes)	67:18	46:8	0.3521	38:10	20:2	0.1792
AST (normal: abnormal)	72:13	52:2	0.0109^*^	37:11	22:0	0.0113^*^
ALT (normal: abnormal)	68:17	48:6	0.0350^*^	33:15	21:1	0.0140^*^
CA19-9 (normal: abnormal)	27:58	36:18	0.0013^*^	20:28	12:10	0.0008^*^
CEA (normal: abnormal)	62:23	51:3	0.0099^*^	27:21	18:4	0.0047^*^
Clinical stage (I/II: III/IV)	33:52	46:8	<0.0001^*^	14:34	20:2	<0.0001^*^
Radiomics score	0.76 ± 0.58	0.09 ± 0.47	< 0.0001^*^	0.78 ± 0.59	0.20 ± 0.60	0.0023^*^

*Individual pre-operative clinical factors are analyzed for significant differences using non-parametric test*.

* *P < 0.05 indicates a significant difference. Maximum diameter, Age and Radiomics score are represented as [mean ± standard deviation]. ER, early recurrence; Non-ER, non-early recurrence; AST, serum aspartate transaminase; ALT, serum alanine transaminase; CA19-9, serum carbohydrate antigen 19-9; CEA, serum carcinoembryonic antigen*.

Six clinical factors showed significant differences between the ER and non-ER groups: maximum tumor diameter, AST level, ALT level, CA19-9 level, CEA level, and clinical stage. Complete R0 resection was obtained in 177 (84.69%) cases of all surgical patients. In the pathologic evaluation of tumor-resection specimens, well-, moderate-, and poorly differentiated tumors were found in 25 (11.96%), 110 (52.63%), and 74 (35.41%) cases, respectively. The power of our test was 0.96, suggesting a sufficient sample size of the validation cohort and a credible conclusion.

### Feature selection, building of a radiomics signature, and validation

**Figure 3:**
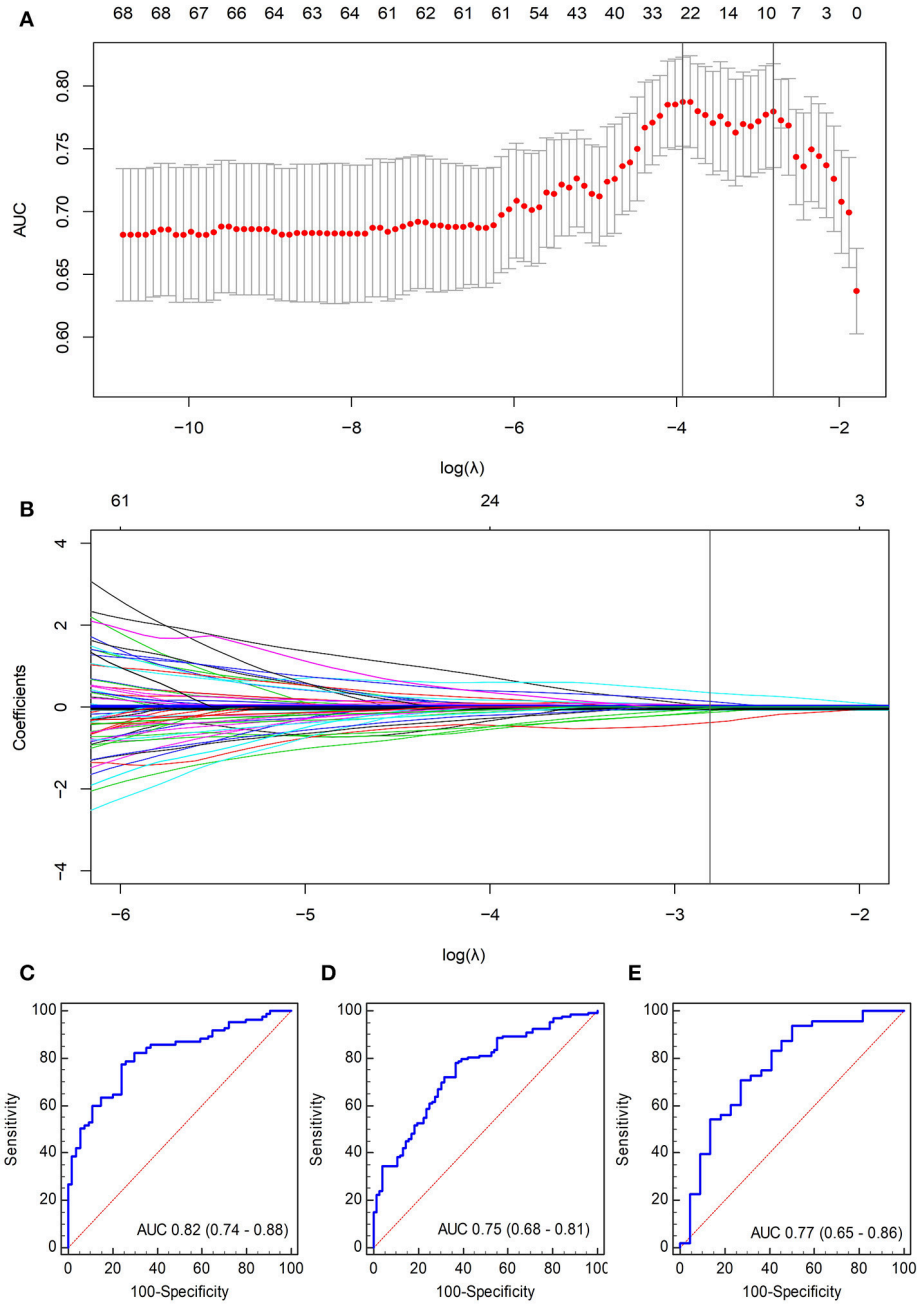
Selection of radiomics features using the LASSO logistic regression algorithm. **(A)** The penalization coefficient λ in the LASSO model was tuned using tenfold cross-validation and the minimum criterion. AUC metrics (y-axis) were plotted against log(λ) (bottom x-axis). The top x-axis indicates the number of predictors for the given log(λ). Red dots indicate average AUC for each model at the given λ, and vertical bars through the red dots show the upper and lower values of the AUC according to the tenfold cross-validation. The vertical black lines define the optimal λ (i.e., where the model provides its best fit to the data). As a result, an optimal λ of 0.0605, with log(λ) = −2.81, was selected. **(B)** LASSO coefficient profiles of the 98 radiomics features. The vertical line was plotted at the given λ, selected by tenfold cross-validation. For the optimal λ, nine features with a non-zero coefficient were selected. ROC curves of the radiomics signature for **(C)** training cohort, **(D)** internal validation cohort, and **(E)** independent validation cohort.

A total of 467 texture features were extracted from arterial-phase images on MRI for each patient. Of these texture features, 98 features showed no significant linear internal correlation. These 98 features were reduced to 9 *via* LASSO regression based on the training cohort (Figures 3A,B). The formula used to calculate the radiomics score (Supplementary Data V) was constructed by linear combination of these nine features multiplied by LASSO coefficients. In general, patients in the ER group showed a significantly higher radiomics score than patients in the non-ER group from the training cohort (0.76 ± 0.34 vs. 0.09 ± 0.22, *p* < 0.001). The AUC of the training cohort was 0.82 (95% confidence interval [CI], 0.74–0.88).

The validation cohorts (i.e., the internal and independent validation datasets) were used to test the prediction power of the radiomics signature. Internal validation was carried out using the data cohort of 209 patients segmented by the second radiologist. Significant differences within radiomics scores were found between the two groups (0.68 ± 0.66 vs. 0.20 ± 0.25, *p* < 0.05) with an AUC of 0.75 (95% CI, 0.68–0.81).

Independent validation was conducted using the data cohort of 70 patients segmented by the first radiologist. The radiomics scores for patients in the ER and non-ER groups were 0.78 ± 0.35 and 0.20 ± 0.36, respectively, and this difference was significant (*p* < 0.05). The AUC for the independent validation was 0.77 (95% CI, 0.65–0.86). The ROC curves are displayed in Figures 3C–E.

### Development, validation, and assessment of the radiomics nomogram

**Figure 4:**
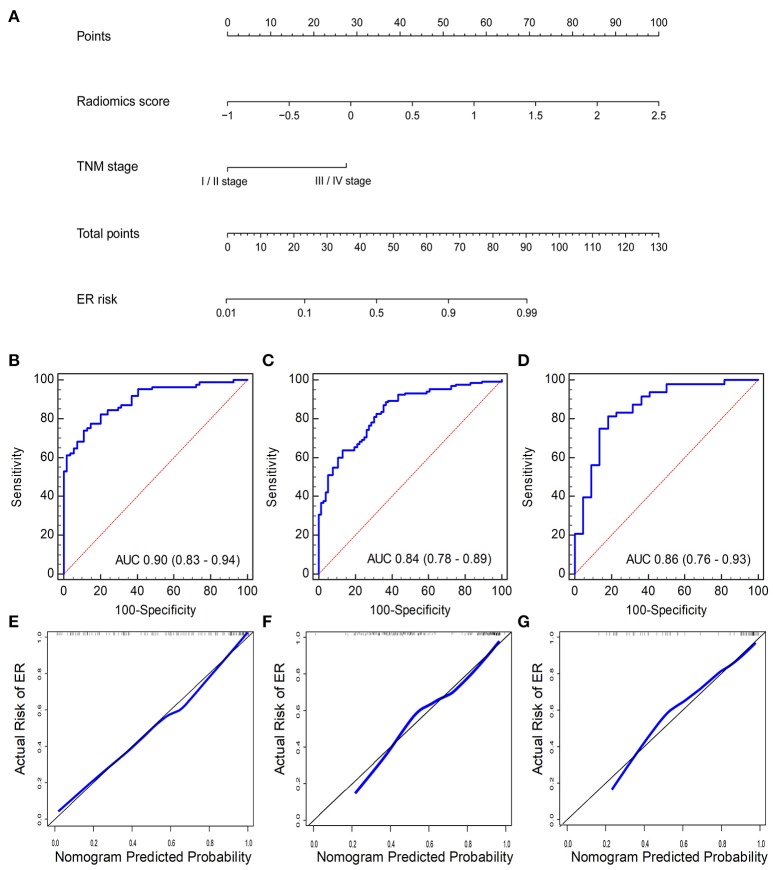
**(A)** Radiomics nomogram combining the radiomics score and clinical stage developed by the training cohort. Performance of the nomogram was assessed by ROC curves and calibration curves in the **(B,E)** training cohort, **(C,F)** internal validation cohort, and **(D,G)** independent validation cohort. Calibration curves describe the calibration of the nomogram with respect to agreement between the predicted risk (x-axis) and real risk (y-axis) of ER. The 45-degree black line represents the “ideal” prediction. The blue line represents the performance of the radiomics nomogram. The blue line closer to the ideal prediction has a higher predictive accuracy of the nomogram.

A logistic regression analysis combining the radiomics signature and clinical stage in the training cohort was conducted. Specific information about the construction of the multivariate logistic regression model can be found in Supplementary Data VI. The model is presented as the nomogram in Figure 4A. The calibration curve of the radiomics nomogram, indicating the probability of ER, showed good agreement between the predicted and observed risks of ER. An H–L test with the training cohort suggested that there was no significant deviation from the ideal fit (*p* = 0.41). The AUC of the nomogram was 0.91 (95% CI, 0.85–0.95, Figure 4B).

Another promising performance was obtained in the internal validation cohort with an AUC of 0.85 (95% CI, 0.80–0.90) (Figure 4C) and a non-significant H–L test statistic (*p* = 0.45). Good performance was also observed with an AUC of 0.88 (95% CI, 0.78–0.94) (Figure 4D) and a non-significant H–L test statistic (*p* = 0.75). The non-significant value of the H-L test statistic suggested no deviation from the perfect predictive model. The calibration curves of the radiomics nomogram are shown in Figures 4E–G. The performance of the radiomics signature and radiomics nomogram is summarized in Table 2.

**Table 2:** Predictive performance of radiomics signature and nomogram.

**Model**	**Radiomics signature**			**Radiomics nomogram**		
	**Sensitivity**	**Specificity**	**AUC (95% CI)**	**Sensitivity**	**Specificity**	**AUC (95% CI)**
Training cohort	0.78	0.76	0.82 (0.74–0.88)	0.74	0.89	0.90 (0.83–0.94)
Internal validation	0.78	0.63	0.75 (0.68–0.81)	0.89	0.64	0.84 (0.78–0.89)
Independent validation	0.94	0.50	0.77 (0.65–0.86)	0.81	0.82	0.86 (0.76–0.93)

*AUC, area under ROC curve; CI, confidence interval*.

**Figure 5:**
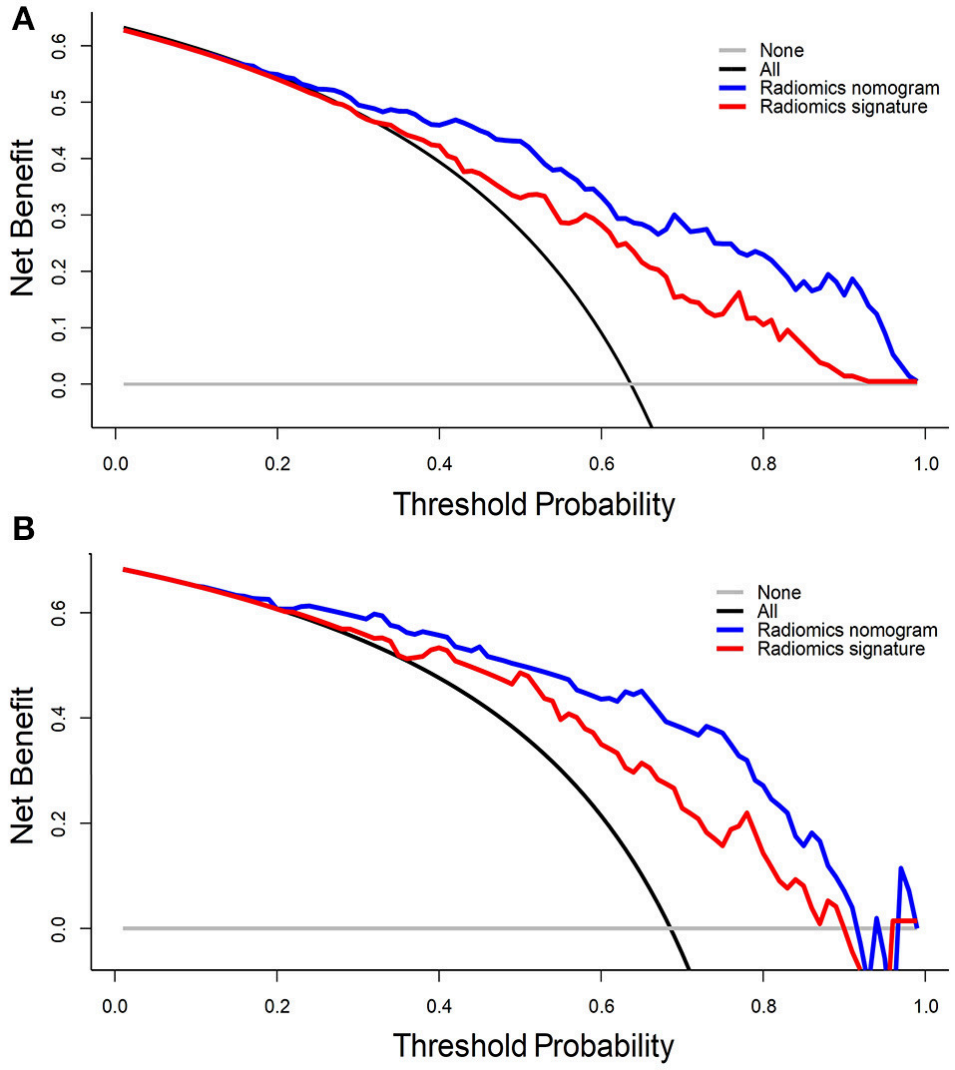
DCA for the radiomics signature and radiomics nomogram in the **(A)** internal validation cohort and **(B)** independent validation cohort. The y-axis represents the net benefit, whereas the x-axis represents the threshold probability. Blue line: radiomics nomogram; red line: radiomics signature; black line: hypothesis that all patients have ER; gray line: hypothesis that no patients have ER.

DCA for the radiomics nomogram model and radiomics signature are presented in Figure 5. For the internal validation cohort, the DCA curve showed that the radiomics nomogram gained more net benefits than the “treat all patients” strategy, the “treat none” strategy, as well as the radiomics signature (range: 0–1). For the independent validation cohort, the DCA curve showed that the nomogram also performed better than the treat-all-patients strategy, the treat-none strategy, and the radiomics signature when the threshold probability for a physician or patient was within a range 0–0.90.

## Discussion

The nomogram described here was constructed using a radiomics signature and clinical stage. The radiomics signature was built *via* radiomics features extracted from MRI scans. The radiomics signature and clinical stage are convenient options to implement in the clinical setting. The radiomics signature was developed using nine features, all of which were extracted from decomposed images (which were decomposed by a three-dimensional wavelet transformation). One could suggest that the wavelet transformation was a multiscale analytical method that could be used to further explore tumor heterogeneity in multiple scales. The wavelet features may also have underlying associations with pathophysiology, proteomics and tumor morphology, which could not be captured by low-level radiomics features or visual inspection by clinicians.

Only two studies have investigated the preoperative prediction of ICC recurrence. Jeong et al. developed a nomogram to predict ICC recurrence after hepatic resection (5). The nomogram was established based on four independent prognostic clinical factors: tumor diameter, Child–Pugh score, lymph-node metastasis, and surface antigen of the hepatitis-B virus level (5). Although this nomogram could predict ICC recurrence, it provided the prediction based only on clinical factors, and had unfavorable accuracy. A study by Ribero et al. established a preoperative model of recurrence scoring based on tumor number, metabolic tumor volume, CEA level, and tumor diameter (25). This scoring model was developed by incorporating information of clinical and functional imaging, but this scoring model could not be used widely because of the high cost of positron emission tomography. Thus, a more feasible method for preoperative prediction of recurrence needs to be investigated.

This is the first study to investigate prediction of ER of ICC using MRI features. In the present study, a radiomics nomogram was developed for preoperative prediction of ER of ICC after partial hepatectomy. The nomogram developed generated a favorable prediction performance by including radiomics features which were highly related to ER in ICC patients.

Whether tumor diameter can serve as an independent risk factor used for the prognosis is controversial. In our study, univariate analysis showed that tumor diameter was associated significantly with ER of ICC. However, tumor diameter was not included in the nomogram because a significant improvement was not observed between the nomogram model with tumor diameter and the model without tumor diameter. Similarly, tumor diameter is excluded as one of the stratification factors in the AJCC staging system (2). Multiple-factor analyses in several ICC studies have reported the lack of a significant relationship between tumor diameter and the prognosis (4, 26). However, other studies have suggested that tumor diameter could be an independent prognostic factor for ICC (5, 27, 28). These differing results may be explained (at least in part) by the heterogeneity of the ICC population.

Whether the serum level of CA19-9 is an independent clinical factor for prediction of ICC recurrence is controversial. Several studies have reported CA19-9 to be an important tumor marker for pre-operative prediction of ICC prognosis (27, 28). However, a recent study excluded the CA19-9 level from a nomogram for predicting ICC recurrence after hepatectomy, and a significant difference was not observed for the relapse-free survival of patients with ICC (5). In the present study, although a significant difference was obtained in the CA19-9 level between patients in the ER and non-ER groups, a significant improvement was not achieved between the nomogram using the CA19-9 level and the model not using the CA19-9 level. Thus, the CA19-9 level was not included in the developed nomogram.

An accurate preoperative prediction of ER could be useful to develop appropriate strategies, particularly because additional chemotherapy would benefit patients at a high risk of ER. In one meta-analysis, ICC patients did not benefit from adjuvant chemotherapy (29). Another study found a similar 5-year recurrence rate and overall survival in ICC patients who had adjuvant transarterial chemoembolization (TACE) compared with non-TACE ICC patients (30, 31). Although those results showed no significant difference in the overall prognosis between chemotherapy and non-chemotherapy groups, ICC patients with high-risk factors were found to benefit from post-hepatectomy adjuvant chemotherapy through stratification of ICC patients with different risks (30, 31). Therefore, ICC patients with high-risk features (e.g., large diameter, multiple tumors, lymphatic involvement) have been suggested to have adjuvant therapy to improve their prognosis in an agreed expert consensus for ICC treatment (32). Currently, there is an absence of objective evaluation criteria/tools for high-risk features in patients with ICC. Our prediction nomogram could provide individualized prediction of short-term recurrence risk, which could be used to stratify patients at high risk of ICC recurrence for adjuvant chemotherapy.

Our study had three main limitations. First, diffusion-weighted MRI data from our institution with different b values over a long-time interval were, regretfully, abandoned in our study because of poor consistency (33). Considering the need for a large cohort in machine learning and convenient application in the future, we used arterial-phase images of contrast-enhanced MRI to extract radiomics features. Second, molecular markers closely correlated with the ICC prognosis were not explored due to a restriction of experimental conditions (34–36). Lastly, this was a single-center retrospective study. Also, all patients were imaged using the same MRI scanner. MRI scans can yield different gray-level ranges between patients because of different scanners, magnetic densities, and acquisition protocols (37–39). Besides, the MRI machine was equipped with an image post-processing function that also impacted the image texture. These differences may impact texture computation. Whether the textures on the MR images are stable between different manufacturers and field strengths is not known. In future work, we will investigate the prediction performance using image data from different centers related to different MR scanners and magnetic densities.

## Conclusions

We built a new nomogram model that combines radiomics features and clinical stage for pre-operative prediction of ER of ICC after partial hepatectomy. This model was validated by a relatively small independent validation cohort. Multicenter retrospective, as well as prospective, validation will be undertaken in subsequent studies to achieve an even higher level of evidence.

## Author contributions

WL, LX, and FC: conception and design. WL, LX, and FC: development of methodology (provided animals, acquired and managed patients, provided facilities, etc.). WL, LX, and PY: analysis and interpretation of data (e.g., statistical analysis, biostatistics, computational analysis). WL, LX, TN, and FC: writing, review, and/or revision of the manuscript. WL, LX, PY, LZ, DW, QH, TN, and FC: administrative, technical, or material support (i.e., reporting or organizing data, constructing databases).

### Conflict of interest statement

The authors declare that the research was conducted in the absence of any commercial or financial relationships that could be construed as a potential conflict of interest.
