# Supplementary material for: Novel Nomogram for Preoperative Prediction of Early Recurrence in Intrahepatic Cholangiocarcinoma
Source: Front Oncol. 2018 Sep 4;8:360. doi: 10.3389/fonc.2018.00360 (PMC6131601; doi:10.3389/fonc.2018.00360)
Supplement: Supplementary file 1 [file Presentation_1.pdf]

Supplementary Material

Novel Nomogram for Preoperative Prediction of Early Recurrence in Intrahepatic Cholangiocarcinoma

Authors

Wenjie Liang [1,2*], Lei Xu[3,4*], Pengfei Yang[3,4], Lele Zhang[5,6,2], Dalong Wan[2], Qiang Huang[1], Tianye Niu[3], and Feng Chen[1†]

***: Both authors shared the first author;**

†: **Corresponding authors:** Feng Chen, chenfenghz@zju.edu.cn

Supplementary Data I:

Recruitment pathway for included patients

Supplementary Data II:

The feature pool developed in this study

Supplementary Data III:

Description of the LASSO algorithm

Supplementary Data IV:

Formula for the radiomics score

Supplementary Data V:

Nomogram for each patient in each cohort

Supplementary Data VI:

Specific information about the construction of the multivariate logistic regression model

I. Recruitment pathway for included patients

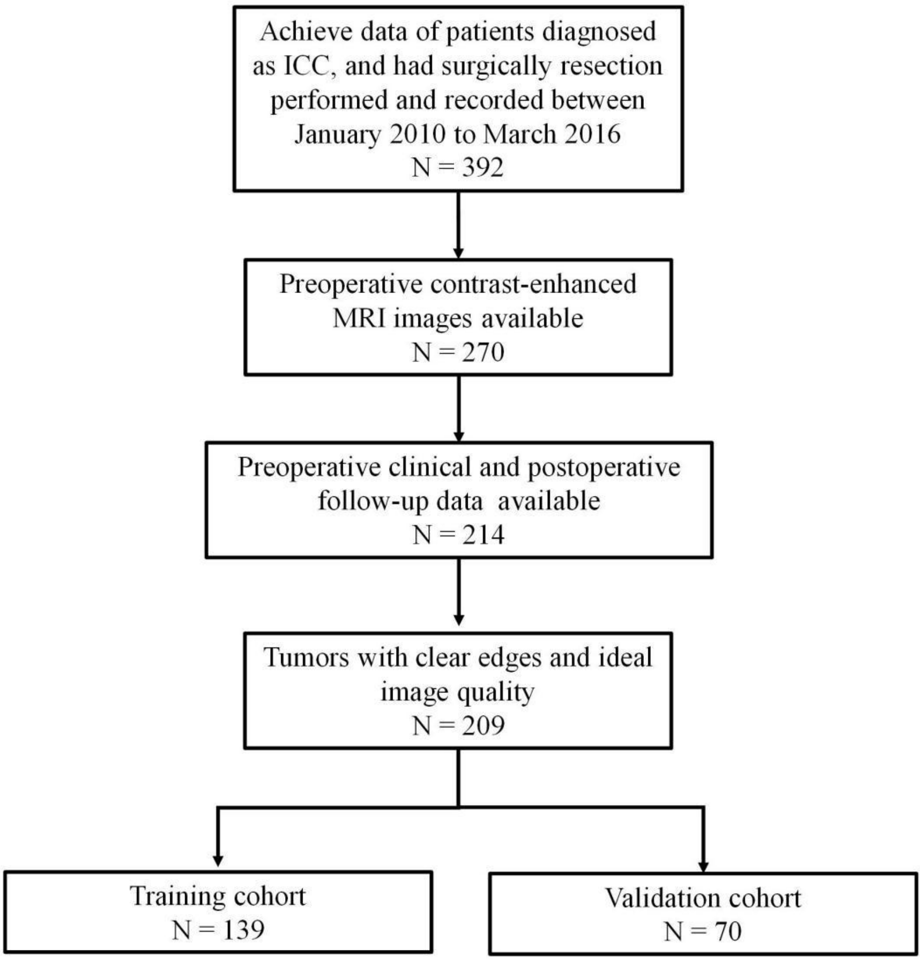

Figure S1. Recruitment pathway for included patients.

II. The feature pool developed in this study

A pool consisting of 467 radiomics features was developed. Features were extracted and organized with MATLAB 2015b (MathWorks, Natick, MA, USA). The 467 features were divided into three types: histogram features, textural features, and features after wavelet transform.

Table S1 Feature pool developed in this study.

**Texture type**	Texture name
**Histogram**	Variance, Skewness, Kurtosis, Mean, Energy, Entropy
**GLCM** (1) **(Grey-level co-occurrence matrix)**	Autocorrelation(autoc), Contrast(contr), Correlation(corrm), Correlation2(corrp), Cluster Prominence(cprom), Cluster Shade(cshad), Dissimilarity(dissi), Energy(energ), Entropy(entro), Homogeneity(homom), Homogeneity2(homop), Maximum probability(maxpr), Sum of squares Variance(sosvh),

	Sum average(savgh), Sum variance(svarh), Sum entropy (out.senth), Difference variance(dvarh), Difference entropy(denth), Information measure of correlation1(inf1h), Informaiton measure of correlation2(inf2h), Inverse difference normalized (INN) (indnc), Inverse difference moment normalized(idmnc)
**GLRLM** (2, 3, 4, 5) **(Grey-level run-length matrix)**	Short Run Emphasis (SRE), Long Run Emphasis (LRE), Grey-Level Non-uniformity (GLN), Run-Length Non-uniformity (RLN), Run Percentage (RP), Low Grey-Level Run Emphasis (LGRE), High Grey-Level Run Emphasis (HGRE), Short Run Low Grey-Level Emphasis (SRLGE), Short Run High Grey-Level Emphasis (SRHGE), Long Run Low Grey-Level Emphasis (LRLGE), Long Run High Grey-Level Emphasis (LRHGE), Grey-Level Variance (GLV), Run-Length Variance (RLV)
**GLSZM** (2, 3, 4, 5) **(Grey-level size zone matrix)**	Small Zone Emphasis (SZE), Large Zone Emphasis (LZE), Grey-Level Non-uniformity (GLN), Zone-Size Non-uniformity (ZSN), Zone Percentage (ZP), Low Grey-Level Zone Emphasis (LGZE), High Grey-Level Zone Emphasis (HGZE), Small Zone Low Grey-Level Emphasis (SZLGE), Small Zone High Grey-Level Emphasis (SZHGE), Large Zone Low Grey-Level Emphasis (LZLGE), Large Zone High Grey-Level Emphasis (LZHGE), Grey-Level Variance (GLV), Zone-Size Variance (ZSV)
**NGTDM** (6) **(Neighbourhood grey-tone difference matrix)**	Coarseness, Contrast, Busyness, Complexity, Strength

Wavelet features: Textural information was decoupled through discrete undecimated 3D wavelet transformation (7) by separating the initial image, in which high-frequency signals were transformed to low-frequency signals. This is pre-processing before feature extraction. Specifically, with the original image X, and low-pass (L) and high-pass (H) wavelet functions, the wavelet decompositions of X were marked as X_LLL, X_LLH, X_LHL, X_LHH, X_HLL, X_HLH, X_HHL, and X_HHH. In this undecimated decomposition, each decomposed image was of equal size to the initial image, and all decompositions were shift-independent. Therefore, decompositions after wavelet transformation could be processed directly by initial description of the gross tumor volume. Histogram and textural features were extracted from each decomposition and resulted in 408 features. Finally, 467 features were extracted from each patient.

Reference:

1. Haralick RM, Shanmugam K. Textural features for image classification. *IEEE Transactions on systems, man, and cybernetics* (1973) 6:610-621.

2. Galloway MM. Texture analysis using gray level run lengths. *Computer graphics and image processing* (1975) 4(2):172-179.

3. Chu A, Sehgal CM, Greenleaf JF. Use of gray value distribution of run lengths for texture analysis. *Pattern Recognition Letters* (1990) 11(6):415-419.

4. Dasarathy BV, Holder EB. Image characterizations based on joint gray level-run length distributions. *Pattern Recognition Letters* (1991) 12(8):497-502.

5. Thibaul G, Fertil B, Navarro C, Pereira S, Cau P, Levy N, et al. Texture Indexes and Gray Level Size Zone Matrix Application to Cell Nuclei Classification. *International Conference on Pattern Recognition and Information Processing* (2009).

6. Amadasun M, King R. Textural features corresponding to textural properties. *IEEE Transactions on systems, man, and Cybernetics* (1989) 19(5):1264-1274.

7. Coroller TP, Grossmann P, Hou Y, Rios Velazquez E, Leijenaar RT, Hermann G, et al. CT-based radiomic signature predicts distant metastasis in lung adenocarcinoma. *Radiother Oncol* (2015) 114(3):345-50.

III. Description of the LASSO algorithm

LASSO (8) is an effective regression method for high-dimensional prediction. LASSO was combined with multifactor logistic regression to predict early recurrence and to select the most prognostic features from the training cohort. LASSO minimizes the log partial likelihood ($l(\beta)$) subjected to the summarized absolute values (β) of the parameters bounded by a constant (s>0):

$$\hat{\beta} = \text{argmin } l(\beta) \text{ subject to } \sum \left|\beta_j\right| \leq s$$

With the benefit from the absolute constraint, LASSO alters the coefficients of most features to zero and selects the features that continue to have non-zero coefficients. Thus, LASSO is feasible for feature reduction and selection of high-dimension data. Here, the standardized constraint parameter was set as 0.06, and nine non-zero coefficients were included in LASSO. Then, the radiomics signature and clinical characteristic were incorporated through multivariable logistic regression.

Before the LASSO test, radiomic features were normalized by subtracting the center and dividing by the root-mean-square of each column. The center and root mean square were computed from the training cohort were used for the training and validation cohorts.

Reference:

8. Tibshirani R. Regression shrinkage and selection via the lasso. *Journal of the Royal Statistical Society Series B (Methodological)* (1996) 55(1):267-88.

IV. Formula for the radiomics score

$$\text{Radiomics score} = 0.496426318 - 0.140041874 * \text{LLL\_GLRLM\_LRE\_DCE} - 0.384249355 * \text{LLL\_GLSZM\_HGZE\_DCE} - 0.102860858 * \text{LHL\_Mean\_DCE} - 0.336343980 * \text{LHL\_GLCM\_energ\_DCE} + 0.044417704 * \text{LHH\_Mean\_DCE} - 0.033780710 * \text{LHH\_GLRLM\_RLV\_DCE} + 0.126088749 * \text{LHH\_GLSZM\_SZE\_DCE} + 0.000893022 * \text{HLH\_GLCM\_cshad\_DCE} + 0.037629843 * \text{HLH\_GLCM\_maxpr\_DCE}$$

V. Nomogram for each patient in each cohort

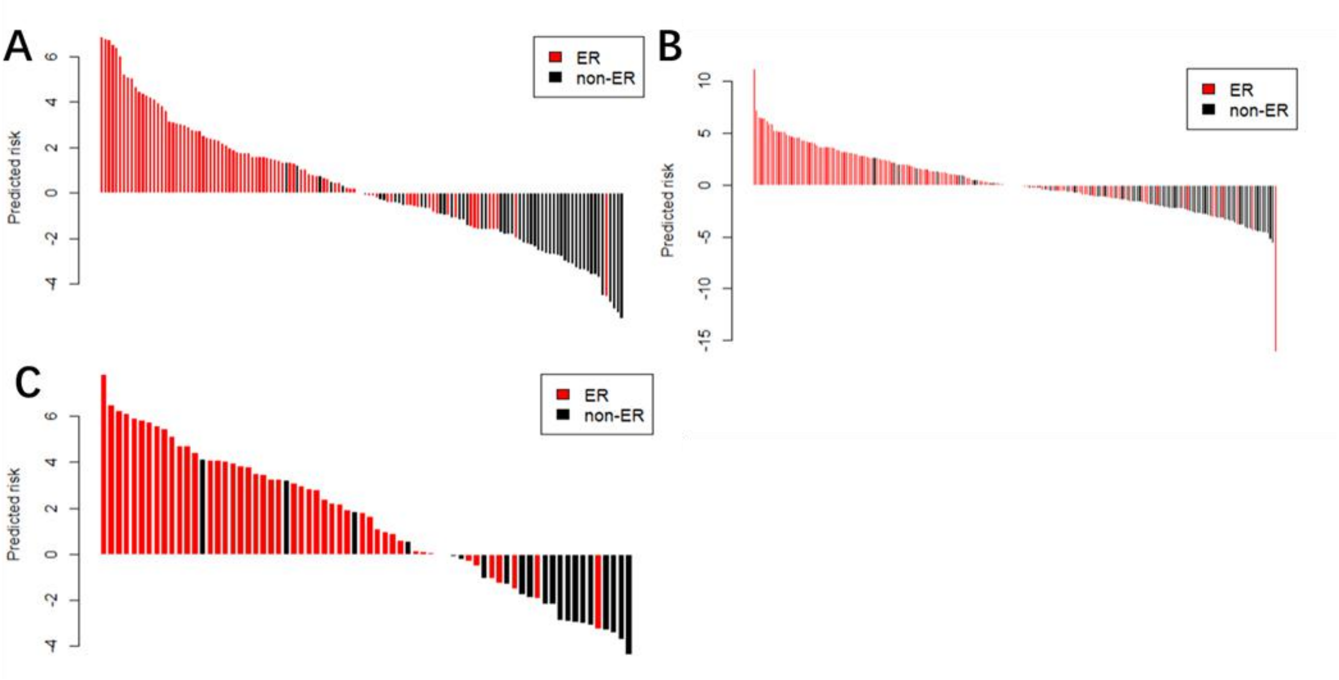

Figure S2. Radiomics nomogram for each patient: (A) training cohort; (B) internal validation cohort; (C) independent validation cohort. The status of ER is marked in different colors.

VI. Specific information about the construction of the multivariate logistic regression model

The multivariate logistic regression model was developed by using the backward search method with the minimum Akaike's information criterion (AIC) criteria. AIC dealt with the trade-off between the goodness of fit and the complexity of the model. The model with minimum AIC demonstrates the best fit and the least free parameters.

The Akaike Information Criterion (AIC) is a relative quality estimator for different models in the same

dataset. The concept of AIC is built the information theory: it estimates the relative information lost when a certain dataset is represented by a model. In this process, it deals with the trade-off between the goodness of fit and the complexity of the model. The purpose of the AIC is to find the optimal model that could best fit the dataset and contain the least free parameters.

Suppose a model for a certain data. Let p indicates the number of free parameters in the model and $\hat{\boldsymbol{L}}$ the likelihood for the model.

Then the AIC is defined as:

$$\boldsymbol{AIC} = -\boldsymbol{2}\ \boldsymbol{ln}\hat{\boldsymbol{L}} + \boldsymbol{2p}$$

For a series of candidate models with the same dataset, the optimal model is the one with the minimum AIC.

The model with minimum AIC demonstrates the best fit and the least free parameters at the same time.
